# Sociodemographic factors associated with IgG and IgM seroprevalence for human cytomegalovirus infection in adult populations of Pakistan: a seroprevalence survey

**DOI:** 10.1186/s12889-016-3772-8

**Published:** 2016-10-22

**Authors:** Saira Ibrahim, Anwar A. Siddiqui, Amna R. Siddiqui, Waquaruddin Ahmed, Paul A. H. Moss, El-Nasir M. A. Lalani

**Affiliations:** 1 Department of Biological and Biomedical Sciences, Aga Khan University, PO Box 3500, Stadium Road, Karachi, 74800 Pakistan; 2 Department of Community Health Sciences, Aga Khan University, PO Box 3500, Stadium Road, Karachi, 74800 Pakistan; 3 Pakistan Medical Research Council, Research Centre, Jinnah Postgraduate Medical Centre, Rafique Shaheed Road, Karachi, Pakistan; 4 Institute of Immunology and Immunotherapy, University of Birmingham, Vincent Drive, Edgbaston, Birmingham, B15 2TT UK; 5 Department of Pathology and Laboratory Medicine, Aga Khan University, PO Box 3500, Stadium Road, Karachi, 74800 Pakistan

**Keywords:** Human cytomegalovirus, Seroprevalence, IgG, IgM, Sociodemographic factors, Pakistan

## Abstract

**Background:**

The seroprevalence of human cytomegalovirus (HCMV) infection ranges from 30 to 90 % in developed countries. Reliable estimates of HCMV seroprevalence are not available for Pakistan. This study determined the seroprevalence and sociodemographic factors associated with HCMV infection in adult populations of Karachi, Pakistan.

**Methods:**

A seroprevalence survey was conducted on 1000 adults, including residents of two semi-urban communities, and visitors to a government and a private hospital. Questionnaire-based interviews were conducted. Sera were analysed for HCMV-specific IgG and IgM. Chi-square or Fisher’s exact test was used for comparing sociodemographic variables against seropositivity of HCMV-IgG or IgM. Multiple logistic regression modeling was performed for IgG seroprevalence and adjusted odds ratios were computed.

**Results:**

The seroprevalence of HCMV-IgG and IgM was 93.2 and 4.3 % respectively. 95.3 % of individuals who were IgM seropositive were also seropositive for IgG. Around 6 % (15/250) of women of childbearing age remained uninfected and were therefore susceptible to primary infection. HCMV-IgG seroprevalence was associated with being female (*p* = 0.001), increasing age (*p* = 0.002) and crowding index (*p* = 0.003) and also with lower levels of both education (*p* < 0.001) and income (*p* = 0.008). Seroprevalence also differed significantly by marital status (*p* = 0.008) and sampling location (*p* < 0.001). A logistic regression model for HCMV-IgG seroprevalence showed associations with being female (OR = 1.89; 95 % CI: 1.10–3.25), increasing age (OR = 3.95; 95 % CI: 1.79–8.71) and decreasing income (OR = 0.72; 95 % CI: 0.54–0.96). A strong association was observed between increased seroprevalence of HCMV-IgM and decreasing household size (*p* = 0.008).

**Conclusions:**

Seroprevalence of HCMV is very high in Pakistan, although 6 % of women of childbearing age remain at risk of primary infection. The IgM seropositivity observed in some individuals living in small household size (1–3 individuals) with persistent HCMV infection could have resulted from a recurrent HCMV infection. Future longitudinal research in pregnant women and neonates is required to study the trends in HCMV seroprevalence over time in Pakistan for the development of a potential HCMV prevention and vaccination programme.

## Background

Human cytomegalovirus (HCMV) is a member of the *Herpesviridae* family, and the virus may be shed intermittently in bodily fluids (saliva, urine, semen, blood and breast milk) [1]. As such, its transmission occurs both horizontally and vertically through close contact and directly from mother to embryo, fetus, or baby [2]. Upon primary infection, which is usually asymptomatic [3], HCMV establishes a state of lifelong latency, during which infectious virus is difficult to isolate [4]. Active HCMV infection can result from primary infection in a previously seronegative individual or reactivation in a seropositive individual [5] in response to immunosuppression and inflammation [6]. Viral reactivation is associated with significant morbidity and mortality in immunocompromised individuals, such as patients with HIV infection or those undergoing solid organ or bone marrow transplantation, and up to 15 % of babies who acquire congenital infection, manifest signs of cytomegalic inclusion disease (CID) at birth [1].

Seroprevalence of HCMV varies from 30 to 90 % in most developed countries [1] and the seroprevalence is dependent on sociodemographic factors [7]. Adult populations in Africa [8], Asia [9–11] and South America [12, 13] have higher HCMV seroprevalence than European [14–16] and North American populations [7]. In addition, seroprevalence is reported with increasing age [7] and an inverse correlation with socioeconomic status [17, 18].

Most HCMV seroepidemiological studies have previously focused on children and women of childbearing age, as they constitute groups at highest risk of developing HCMV infection [19, 20]. Studies determining seroprevalence of HCMV-IgG antibodies in the general population are predominantly limited to developed countries that have assessed the impact of sociodemographic factors on HCMV-IgG seropositivity [7, 18]. Similar research in developing countries are lacking in terms of sample size and in depth analysis of sociodemographic data [9, 12]. Moreover, the sociodemographic characteristics of HCMV-IgM seroprevalence have not been widely explored both in developed and developing countries.

At the current time, there is minimal information regarding the epidemiological determinants of HCMV infection in Pakistan [21]. We undertook a study to determine the seroprevalence of HCMV-specific IgG and IgM antibodies and also to identify the sociodemographic factors associated with HCMV-IgG and HCMV-IgM seropositivity in adult populations of Karachi, Pakistan.

## Methods

### Study design and locations

We conducted a seroprevalence survey during the period from July 2010 to June 2012 in adult populations of Karachi, Pakistan. Study locations comprised of two major hospitals and two medical camps held in two semi-urban communities. The hospital location comprised of gastroenterology sections of two major tertiary care government and private hospitals, namely, Jinnah Postgraduate Medical Centre (JPMC) and Aga Khan University Hospital (AKUH) respectively. The Department of Gastroenterology and Hepatology unit at JPMC serves as a screening centre for patients from across Pakistan suspected of being infected with hepatitis B or C. The section of gastroenterology at AKUH provides high quality standard care for liver and pancreato-biliary diseases and also serves as a referral centre for interventional procedures to treat gastrointestinal ailments. Visitors to JPMC are predominantly from a lower socioeconomic class whilst most patients attending AKUH are of middle to high socioeconomic status.

Karachi is a metropolitan city with six districts, divided into 18 towns [22]. It has a multi-ethnic and multi-lingual population, majority being Sindhi and Urdu speaking. Two semi-urban communities, namely, Jam Goth and Radho Jokhio Goth were selected from Malir town and Gadap town respectively, located in Malir district, Karachi. Area wise, Gadap town is larger than Malir town, the latter being more densely populated. Gadap town is amongst the least developed areas of Karachi whilst Malir town is relatively more developed. Both towns are inhabited by ethnically diverse communities.

### Study population and sampling

All consenting males and females (*N* = 1000) aged 18 years or older at the time of enrolment were included in this study. The study population comprised of patients visiting the gastroenterology sections of JPMC and AKUH, and people residing in two semi-urban communities; and for analysis purpose identified as community A (Malir town) and community B (Gadap town). The sample size estimate was based on the assumption that approximately 80 % of the population would be seropositive for HCMV [23]. An alpha level of 0.05, margin of error of 3.5 %, and design effect of 2 was used for sample size calculation.

Participants from the two towns were approached after consultation, discussion, engagement and agreement of community leaders with whom the aims of our study were explained. In all sampling locations, a convenient sampling technique was employed to select the study participants. The study objectives were explained to the study participants in the simplest possible manner to satisfy the understanding of the respondents. A blood sample was collected and a questionnaire-based interview was conducted with all consenting participants.

### Questionnaire and data collection

The questionnaire-based interviews were designed to obtain personal and sociodemographic information of the participants. The contents of the questionnaire were in compliance with previous studies conducted to determine associated factors of HCMV infection [7, 17]. The questionnaire was developed in English and translated into Urdu (the national language of Pakistan). The English and Urdu versions were pre-tested by conducting in-person interviews of the staff at the AKUH and residents of community A respectively and both versions were modified accordingly.

The sociodemographic characteristics of the participants were assessed by a set of variables, namely, age (years), gender, marital status (married/unmarried/divorced/widowed/separated), ethnicity defined by mother tongue (Sindhi/Baloch/Punjabi/Pakhtoon/Urdu speaking/Other), education level (years of school attended), house construction material (straw or wood or mud/mix of mud and concrete bricks/concrete bricks only), monthly household income was asked in local currency of Rupees, and for analysis purpose was converted as equivalent to U.S. dollars (USD), number of rooms in the house and household (HH) size that was defined as total number of people living together in the same house; the last two variables were used as indicators of crowding to create a new variable ‘crowding index’ (total number of residents per household divided by the total number of rooms in house). Continuous variables for age, income, household size and crowding index when categorised were done using quartiles. Some variables were categorised into two categories if sparse data were available, for example, few participants were either divorced, widowed or separated, then the marital status was grouped into two categories of married and unmarried.

### Laboratory analysis

Samples of up to five ml of blood were collected in serum separation tubes (BD Biosciences, San Jose, CA, USA). Serum was separated and analysed for the presence of HCMV-IgG or IgM in duplicate using commercial enzyme immunoassay kits (BioCheck Inc, Foster City, CA, USA). Absorbance was recorded at 450 nm within 15 min on a Chameleon Microplate Reader (Hidex, Turku, Finland). Positive and negative results of the assays were determined according to the manufacturer’s specifications.

### Statistical analysis

Data entry and statistical analysis were performed using SPSS version 19.0 (IBM Corporation, Chicago, IL, USA). Continuous variables were checked for normal distribution and log transformation was attempted to improve the distribution and when necessary were categorised according to median and quartiles. The outcome variables were the positive or negative sero-status of HCMV-IgG and IgM. Cross tabulations were performed against the sociodemographic variables. Chi-square or Fisher’s exact test was used as a statistical test of significance for comparing sociodemographic variables against seropositivity of HCMV-IgG or IgM. A *p* value of < 0.05 from two-tailed tests was considered significant in all tests. Odds ratios (OR) and 95 % confidence intervals (CI) were calculated for the relationships between sociodemographic variables and seropositive status for HCMV-IgG or IgM. Multiple logistic regression modeling was performed for IgG seroprevalence and adjusted ORs were computed.

## Results

Of the 1000 participants, 63.4 % were enrolled at the government hospital and nearly 14.9 % at the private hospital whilst 3.6 % in community A and 18.1 % in community B were recruited. The study group comprised of 46.3 % males and 53.7 % females with a mean age of 36 years (standard deviation [SD] = 12.6). The mean age of males and females was 35.3 years (SD = 13) and 36.6 years (SD = 12.3) respectively. There were more than six ethnic groups; Sindhi (30.1 %), Baloch (9.9 %), Punjabi (11.9 %), Pakhtoon (14.1 %), Urdu speaking (19.9 %), and ‘Other’ (14.1 %). Overall, 36.8 % of the participants were not literate whilst 13.8, 23.7 and 25.7 % reported 1-6, 7-10 and > 10 years of education respectively. The majority of participants (66.6 %) lived in concrete houses compared with 33.4 % inhabiting houses made of straw/wood/mud or mix of mud and concrete. Only 9.1 % of individuals lived in houses with ≤ 3 persons per household. The distribution of household crowding index of participants was ≤ 2 (28.9 %), > 2-3 (20.9 %), > 3-5 (27.5 %), and > 5 (22.7 %). Monthly income varied from ≤ USD 70 in 30.6 %, USD 71–100 in 21.1 %, USD 101–200 in 25.3 %, and > USD 200 in 23 % (Table 1).

**Table 1:** Seroprevalence of anti-HCMV IgG antibodies in relation to sociodemographic characteristics of the study population

Characteristic	Total	IgG^+^	IgG^−^	OR (95 % CI)	p*
	*N* = 1000	*n* = 932	*n* = 68		
	%	(%)	(%)		
Sampling location					<0.001
Private hospital	14.9	125 (13.4)	24 (35.3)	1.0	
Government hospital	63.4	602 (64.6)	32 (47.1)	3.61 (1.98–6.57)	
Community A	3.6	31 (3.3)	5 (7.4)	0.20 (0.04–0.92)	
Community B	18.1	174 (18.7)	7 (10.3)	4.77 (1.88–12.60)	
Gender					0.001
Female	53.7	514 (55.2)	23 (33.8)	2.41 (1.39–4.18)	
Male	46.3	418 (44.8)	45 (66.2)	1.0	
Age (years)^a^					0.002
≤ 25	26.1	232 (24.9)	28 (41.2)	1.0	
26–34	24.3	222 (23.8)	20 (29.4)	1.34 (0.71–2.55)	
35–45	27.7	268 (28.8)	8 (11.8)	4.04 (1.72–9.84)	
> 45	22.0	207 (22.2)	12 (17.6)	2.08 (0.99–4.46)	
Marital status					0.008
Married	76.5	722 (77.5)	43 (63.2)	2.0 (1.16–3.45)	
Unmarried	23.5	210 (22.5)	25 (36.8)	1.0	
Ethnicity					0.34
Sindhi	30.1	281 (30.2)	20 (29.4)	0.49 (0.14–1.56)	
Baloch	9.9	91 (9.8)	8 (11.8)	0.40 (0.10–1.51)	
Punjabi	11.9	115 (12.3)	4 (5.9)	1.0	
Pakhtoon	14.1	131 (14.1)	10 (14.7)	0.46 (0.12–1.64)	
Urdu speaking	19.9	180 (19.3)	19 (27.9)	0.33 (0.09–1.06)	
Other	14.1	134 (14.4)	7 (10.3)	0.67 (0.16–2.61)	
Education level (years of schooling)					<0.001
0	36.8	356 (38.2)	12 (17.6)	4.52 (2.20–9.45)	
1–6	13.8	133 (14.3)	5 (7.4)	4.06 (1.47–12.11)	
7–10	23.7	220 (23.6)	17 (25.0)	1.97 (1.03–3.80)	
> 10	25.7	223 (23.9)	34 (50.0)	1.0	
Household size (no. of persons)^a^					0.98
1–3	9.1	83 (9.0)	7 (10.3)	1.0	
4–6	31.2	289 (31.3)	20 (29.4)	1.22 (0.45–3.18)	
7–10	36.9	340 (36.9)	25 (36.8)	1.04 (0.51–2.07)	
> 10	22.8	210 (22.8)	16 (23.5)	1.11 (0.40–2.99)	
Household income (USD)					0.008
≤ 70	30.6	291 (31.2)	15 (22.1)	2.58 (1.28–5.23)	
71–100	21.1	198 (21.2)	13 (19.1)	2.03 (0.97–4.28)	
101–200	25.3	240 (25.8)	13 (19.1)	2.46 (1.18–5.17)	
> 200	23.0	203 (21.8)	27 (39.7)	1.0	
House construction material^a^					0.79
Concrete bricks only	66.6	619 (66.5)	46 (67.6)	1.0	
Mud and concrete mix	14.4	136 (14.6)	8 (11.8)	1.26 (0.56–2.97)	
Straw/wood/mud	19.0	176 (18.9)	14 (20.6)	0.93 (0.48–1.83)	
Crowding index^a^					0.003
≤ 2	28.9	253 (27.4)	33 (48.5)	1.0	
> 2–3	20.9	197 (21.4)	10 (14.7)	2.57 (1.18–5.72)	
> 3–5	27.5	258 (28.0)	14 (20.6)	2.40 (1.21–4.84)	
> 5	22.7	213 (23.1)	11 (16.2)	2.53 (1.19–5.45)	

The adjusted OR and 95 % CI were calculated

*USD* U.S. dollar, *OR* odds ratio, *CI* confidence interval

*Comparisons between HCMV-IgG seroprevalence and sociodemographic characteristics were performed by Chi-square or Fisher’s exact test (*p* < 0.05)

^a^The total sample size (n) for these variables may not equal the total number of samples (*N* = 1000) because information was unavailable

**Fig. 1:**
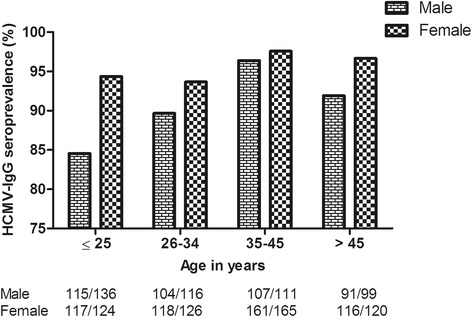
Patterns of HCMV-IgG seroprevalence across different age groups in males (*n* = 462) and females (*n* = 535). The numbers below each bar represent seropositive males and females in each corresponding age group

The seroprevalence of HCMV-IgG was 93.2 % (95 % confidence interval [CI]: 91.5–94.6). The seroprevalence of IgG differed by sampling location, as there were 83.9 % (125/149) from private hospital visitors, 95 % (602/634) from government hospital visitors, 86.1 % (31/36) from community A residents, and 96.1 % (174/181) from community B residents. Using private hospital as reference, government hospital and community B participants had greater odds of being IgG positive. Similarly, females and married persons had greater odds of being IgG positive than males and unmarried persons respectively. Older age groups had higher prevalence of IgG and younger age groups displayed more IgG seronegativity (Table 1). Over 80 % of males and females were already infected with HCMV by the age of ≤ 25 years. However, seroprevalence varied by a 10 % difference between males (84.6 %) and females (94.4 %) in this age group. Seroprevalence in females remained consistently high during ageing and peaked at over 97 %. Within men, seroprevalence increased from 84.6 % (<25 years) to 96.4 % (35–45 years) with age. Around 6 % (15/250) of women aged < 35 years, considered to be prime child bearing age in Pakistan, remained IgG seronegative when primary infection within men of corresponding age was 219/252 (86.9 %). As such, 10 % of men underwent viral seroconversion over a 20 year period, indicating a rate of primary infection of around 0.5 % per year (Fig. 1). An individual’s level of education had a significant effect on the odds of being IgG positive, with up to 6 years of education increasing the odds four times. Lower levels of monthly income had greater odds of being associated with higher IgG seroprevalence compared to highest level of monthly income. Increased crowding index was associated with greater odds of having IgG positive test (Table 1).

The overall seroprevalence of IgM was 4.3 % (43/1000, 95 % CI: 3.2–5.7 %). Forty one individuals (41/1000, 4.1 %) were HCMV-IgM and HCMV-IgG seropositive, whilst only 2 (2/1000, 0.2 %) were HCMV-IgM seropositive and HCMV-IgG seronegative. 95.3 % (41/43) of individuals who were IgM seropositive were also seropositive for IgG. Sixty six samples (6.6 %) were found to be seronegative for HCMV. Smaller household size for number of persons was strongly associated with a higher seroprevalence of IgM (*p* = 0.008). Using reference of HH size of > 10 persons, HH size of 1–3 persons had 23.3 % as IgM positive compared to 8.4 % IgM negative (OR = 4.58 95 % CI: 1.47–14.72); HH size of 4–6 persons had 25.6 % as IgM positive compared to 31.5 % IgM negative (OR = 1.35 95 % CI: 0.45–4.18); HH size of 7–10 persons had 37.2 % IgM positive compared to 36.9 % IgM negative (OR = 1.68 95 % CI: 0.61–4.89); with an overall adjusted OR of 2.1 (95 % CI: 1.2–4.2). The seroprevalence of IgM did not differ significantly by other variables.

In multivariable analysis adjusting for sampling location, the odds of HCMV infection were nearly twice as high for females compared to males (OR = 1.89 95 % CI: 1.10–3.25). Increasing income on a log-transformed scale had lower odds of being positive for HCMV infection (OR = 0.72 95 % CI: 0.54–0.96). Increasing age on a log-transformed scale was associated with greater odds of seropositivity for HCMV (OR = 3.95 95 % CI: 1.79–8.71) (Table 2). Univariate analysis showed that “married” marital status was significantly associated (*p* = 0.008) with HCMV-IgG seroprevalence, which was not observed when we undertook multiple logistic regression analysis in the presence of increasing age and other variables.

**Table 2:** Multiple logistic regression model for determinants of HCMV-IgG seropositivity

Characteristic	β value (S.E.)	Adjusted OR (95 % CI)
Gender		
Female	0.63 (0.27)	1.89 (1.10–3.25)
Male (reference)		
Household income in USD (log-transformed)	−0.31 (0.14)	0.72 (0.54–0.96)
Age in years (log-transformed)	1.37 (0.40)	3.95 (1.79–8.71)
Sampling location		
Private hospital (reference)		
Government hospital	0.90 (0.37)	2.46 (1.18–5.13)
Community A	−0.50 (0.65)	0.60 (0.16–2.19)
Community B	1.15 (0.51)	3.16 (1.14–8.71)

The adjusted OR and 95 % CI were calculated

*USD* U.S. dollar, *S.E.* standard error, *OR* odds ratio, *CI* confidence interval

## Discussion

In this study, we determined the seroprevalence of HCMV infection in adult populations of Pakistan. Our results reveal a high seroprevalence of HCMV infection, with an average of 93.2 % of individuals aged ≥ 18 years, showing the presence of HCMV-specific IgG antibodies indicative of persistent infection. This is consistent with studies reported from Asia, South America and the Caribbean [10, 12, 24] but higher than that reported from North America, Europe, Australia and Africa [7, 8, 15, 16, 25]. The IgM seroprevalence was 4.3 %, which is similar to a previously reported estimate [26] but higher than 0.9–3 % IgM seroprevalence in several other countries [11, 12, 27–30]. However, much greater variations in serum IgM seroprevalence (0–19.5 %) have been reported from developing countries [9, 31]. 95.3 % of individuals who were IgM seropositive were also seropositive for IgG. In the absence of either a confirmed sero-conversion or IgG avidity testing it was not possible to define what proportion of these cases represented primary infection or non-primary reactivation of IgM. Interestingly, a recent analysis of HCMV IgM seroprevalence in women of reproductive age in USA also found that 97.5 % of IgM seropositive women were CMV IgG seropositive [32]. IgG avidity testing in countries with a high prevalence of HCMV infection, such as Korea and Turkey; have shown that none of the women with an IgM seropositive and IgG seropositive sero-status had evidence of a primary infection [33, 34]. As such, we would suggest that the great majority of IgM seropositive cases within our cohort represent viral recurrent reinfection or reactivation rather than primary infection.

Despite the general very high seroprevalence of HCMV infection, 6.6 % of the population remained seronegative. It is likely that good hygiene, hand washing and limited sharing of edibles and used utensils serve to limit infection rates in some populations [35]. The low percentage of individuals with a HCMV-IgM positive and HCMV-IgG negative profile (0.2 %) indicates that the great majority of infections occur during childhood although our data also reveal a significant increase in seroprevalence of men between the ages of 25 and 34 years.

Significant differences apparent in relation to the seroprevalence of HCMV infection in different locations are likely to be attributed to the marked differences in socioeconomic status between the government and private hospital patient populations. In particular, a very high proportion of the patients visiting JPMC belong to the low socioeconomic strata of the society in whom HCMV-IgG seroprevalence reached 95 %.

When we compared the HCMV-IgG seropositivity between the different age groups, we observed a rise in HCMV-IgG seropositivity from age group ≤ 25 years to age group 35–45 years. However, this increase is not as high as has been observed in previous studies and reflects the high baseline prevalence of infection by the time of early adulthood [16, 36]. Nevertheless, our findings concur with other studies from areas of high seroprevalence where a positive HCMV-IgG response was observed in more than 90 % of the individuals in the age group 18 to 35 years [9, 12].

Gender differences in HCMV seroprevalence have been noted previously [7, 15, 16, 36]. In our study, younger adult males are shown to be much more vulnerable to a primary HCMV infection than females and seroprevalence within men increased by 12 % between the age groups ≤ 25 years and 35–45 years. Women can acquire primary infection through their own infected children’s urine and saliva [37]. Therefore those women who remain HCMV seronegative as they enter early adulthood are more likely to be at risk of acquiring HCMV infection from their children than men at this stage. Importantly, nearly all women have had a primary HCMV infection as they reach childbearing age in contrast to developed countries where many women remain seronegative at reproductive age [15, 16, 25]. This is important in relation to the potential susceptibility to primary infection during pregnancy as this is associated with risk of congenital transmission. The seroprevalence of HCMV infection remained high in women of all ages and was still found in 96 % of those aged over 45 years. Interestingly, the seroprevalence of HCMV was slightly reduced in men aged > 45 years. Reasons for this age-related reduction are unclear. It is very unlikely that seroprevalence of HCMV was lower during early adulthood in the current group of individuals aged > 45 years. As such, it is possible that HCMV infection has been associated with excess mortality within males, such that the proportion of HCMV seronegative individuals may have increased slightly over time. Indeed, infection has regularly been correlated with a significant increase in mortality rates in older individuals and it is possible that this effect is more pronounced within male subjects in developing countries.

Previous studies examining the relation between HCMV seroprevalence and socioeconomic status in developing countries have examined small sample size compared to studies undertaken in developed countries [7, 18, 38]. By targeting a relatively large population, we were able to demonstrate an inverse relationship between socioeconomic status and HCMV-IgG seroprevalence, which is consistent with several previous reports [15, 17, 18, 36, 39]. However, in contrast to a previous study [7], household size does not seem to contribute to the disparity in persistent HCMV infection within our population. It has been suggested that people at the lower ends of the education and income spectrum are likely to spend longer time with HCMV infection [17], primarily due to more crowded living conditions [40]. However the definitive reason for the increased seroprevalence of HCMV within poorer communities remains unclear.

Univariate analysis showed a significant association between ‘married’ marital status and HCMV IgG seroprevalence, which is reported to reflect increased viral exposure via vertical and horizontal modes of transmission [37, 41]. The prevalence of HCMV infection did not appear to be influenced by ethnicity in our population.

A unique feature of our study was an epidemiological assessment of sociodemographic factors in relation to IgM sero-status as this has not been widely studied previously. An important finding was that only two individuals were IgM seropositive but IgG seronegative, a pattern which implies recent primary infection. However, false positive HCMV IgM results due to interfering infections in IgG seronegative individuals may exist. As such, as discussed above, IgM sero-status is likely to reflect a humoral response to viral reinfection or reactivation [28, 42]. Indeed, HCMV infection is unusual in that IgM seropositivity can occur in individuals with persistent infection, whereas an IgM antibody response is generally observed only in the setting of acute infection for other pathogens. A striking observation was the increased seroprevalence of HCMV-IgM in association with decreasing household size. No association was seen between IgM and increasing crowding index and so increased frequency of HCMV transmission due to close contact with infected individuals is unlikely to be an explanation. Indeed our data may suggest an opposite explanation, in that smaller families are less likely, at any given time, to house an individual with primary infection. As such, it may be that the regular environmental exposure to HCMV in larger families serves to boost established HCMV-specific immunity and control endogenous viral replication. In this regard, individuals who do not get such regular environmental challenge may undergo more frequent episodes of endogenous viral replication, which would stimulate recurrent IgM immune responses. Interestingly, a recent study of IgM seroprevalence in US women observed a non-significant increase in prevalence within single women compared to married women (6.8 % vs 3.8 %) which could be compatible with this observation [32]. Further epidemiological and virological studies will be required to investigate this observation further.

The two major strengths of this study included a large sample size (*N* = 1000) and first estimate for HCMV seroprevalence in diverse subgroups of population in a developing country. Our comprehensive analysis was based on a questionnaire which gathered data about living conditions and provided an insight into the reasons that could account for such a high seroprevalence of this virus in a developing country.

Congenital infection with HCMV is a significant cause of morbidity and mortality and several candidate HCMV vaccines are under investigation. However, the development of a vaccine programme must take into account the potential risk of increasing the proportion of women who remain HCMV seronegative as they enter pregnancy. Our findings reveal that up to 6 % of women of childbearing age remain HCMV seronegative in Pakistan, and the risk of primary infection within this group may be significant given our observation that around 0.5 % of men of a similar age may be undergoing primary infection each year, an event followed by prolonged viral secretion, which could serve to drive infection in the women who remain uninfected at this age.

Our study serves as a preliminary work towards improved understanding about the status of HCMV infection in Pakistan; mainly so because such a large number of participants with sociodemographic data has not been studied for the assessment of this particular virus.

### Limitations

Some limitations of the work include the study design and broad age categories, which prevented modeling of a definitive temporal relationship between age and HCMV seroprevalence.

## Conclusions

In conclusion, this study suggests that the seroprevalence of HCMV infection varies considerably by gender, age, marital status and socioeconomic status in this setting in Pakistan. Despite the high overall prevalence of HCMV infection, around 6 % of women of childbearing age remained at risk of primary infection. Amongst those with persistent infection, people who live in smaller households are more likely to demonstrate IgM seropositivity. IgM production is likely to indicate a response to endogenous viral reactivation and suggests that the more frequent exposure to viral infection within larger household size may boost protective endogenous immunity.

More research is needed to study and understand the time trends in the epidemiology of HCMV infection among seronegative pregnant women and neonates in the high endemic population of Pakistan that would lead to the initiation of a potential HCMV prevention and vaccination programme when available.

## Abbreviations

AKUH: Aga Khan University Hospital
CI: Confidence interval
CID: Cytomegalic inclusion disease
HCMV: Human cytomegalovirus
HH: Household
JPMC: Jinnah Postgraduate Medical Centre
OR: Odds ratio
SD: Standard deviation
USD: U.S. dollar.
